# Supplementary material for: Acrolein Induces Retinal Abnormalities of Alzheimer’s Disease in Mice
Source: Int J Mol Sci. 2023 Sep 1;24(17):13576. doi: 10.3390/ijms241713576 (PMC10487815; doi:10.3390/ijms241713576)
Supplement: Supplementary file 1 [file ijms-24-13576-s001.zip › ijms-2538857-supplementary.pdf]

International Journal of
Molecular Sciences

MDPI

Supplementary Materials:

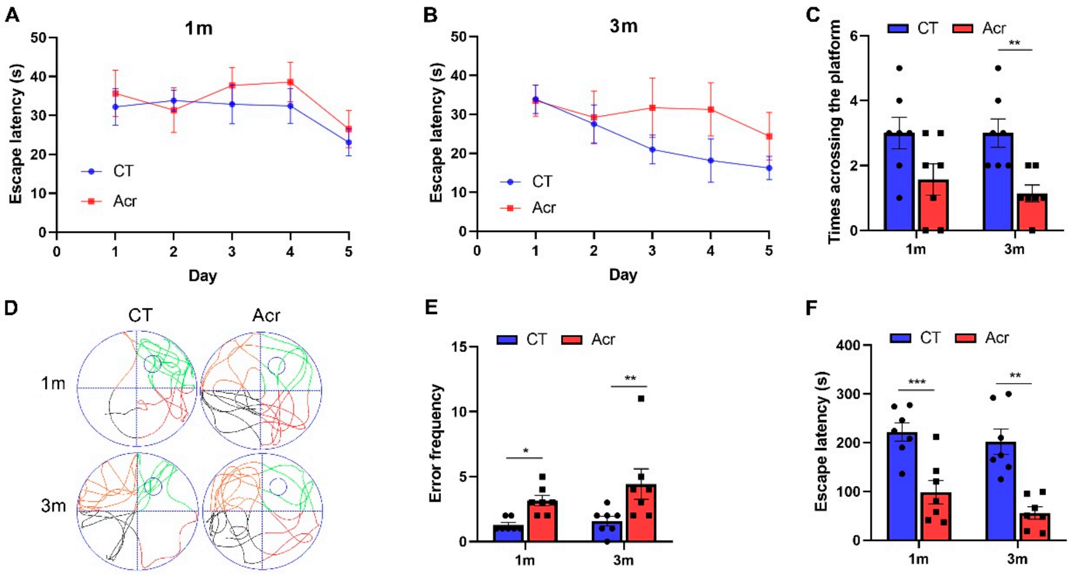

Figure S1. Acrolein induced learning and memory impairment in mice. Escape latencies of the mice in the hidden platform tests, which were conducted for five consecutive days in the (A) 1 and (B) 3 months groups. (C) Number of crossing through the targeted quadrant in the probe test. (D) Swimming tracks of the mice in the probe tests. (E) Error frequency of entering the dark room in the step-through test . (F) Escape latency of entering the dark room. n = 7 for each group. Student's *t*-test was used. Data are presented as mean ± SEM. *$p < 0.05$, **$p < 0.01$, and ***$p < 0.001$ vs. control.

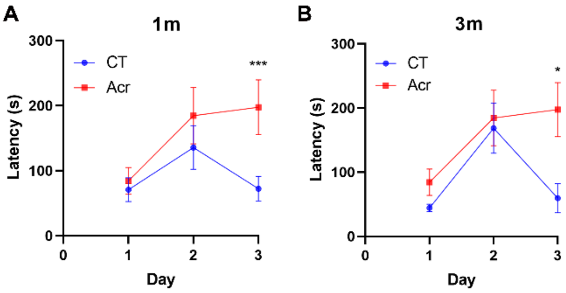

Figure S2. Acrolein induced olfactory impairment in mice in BFPT. Escape latency of mice for the (A) 1 and (B) 3 months groups, which were conducted for three consecutive days. $n = 7$ for each group. Student's *t*-test was used. Data are presented as mean ± SEM. * $p < 0.05$, *** $p < 0.001$ vs. control.
